# Long-term burden of war injuries among civilians in LMICs: case of the July 2006 war in Lebanon

**DOI:** 10.3389/fpubh.2023.1305021

**Published:** 2023-12-08

**Authors:** Elsa Kobeissi, Marilyne Menassa, Gladys Honein-AbouHaidar, Nassim El Achi, Zahi Abdul-Sater, Theresa Farhat, Dalia Al Mohtar, Marwan Hajjar, Rima A. Abdul-Khalek, Bachar F. Chaya, Ahmad Elamine, Shehan Hettiaratchy, Ghassan Abu-Sittah

**Affiliations:** ^1^Conflict Medicine Program, Global Health Institute, American University of Beirut, Beirut, Lebanon; ^2^Refugee Health Program, Global Health Institute, American University of Beirut, Beirut, Lebanon; ^3^Hariri School of Nursing, American University of Beirut, Beirut, Lebanon; ^4^Department of Surgery, Division of Plastic and Reconstructive Surgery, American University of Beirut Medical Centre, Beirut, Lebanon; ^5^Faculty of Health Sciences, American University of Beirut, Beirut, Lebanon; ^6^Department of Surgery and Cancer, Imperial College London, London, United Kingdom; ^7^St Mary's Hospital, Imperial College Healthcare NHS Trust, London, United Kingdom

**Keywords:** armed conflict, Lebanon, long-term burden, access to health care, patient referral, blast injuries, quality of life

## Abstract

**Introduction:**

Lebanon, a country located on the eastern shore of the Mediterranean Sea, is one of the world’s smaller sovereign states. In the past few decades, Lebanon endured a perpetual political turmoil and several armed conflicts. July 12, 2006, marked the start of a one-month war in Lebanon, which resulted in thousands of casualties. Little is known about the long-term consequences of war injuries inflicted on civilians during the July 2006 war.

**Methods:**

The objectives of this paper were to identify and evaluate: 1- civilians’ access to healthcare and medicine under conditions of war; 2- the long-term socioeconomic burden on injured civilians; and 3- their quality of life more than a decade post-war. We adopted a mixed-method research design with an emphasis on the qualitative component. We conducted interviews with patients, collected clinical and financial data from hospital medical records, and administered a self-rated health questionnaire, the EQ-5D-5L. Simple descriptive statistics were calculated using Excel. NVivo 12® was used for data management and thematic analysis.

**Results:**

We conducted 25 interviews. Injured civilians were mostly males, average age of 27. The most common mechanism of injury was blast injury. Most patients underwent multiple surgeries as well as revision surgeries. The thematic analysis revealed three themes: 1- recall of the time of the incident, the thousand miles journey, and patients’ access to services; 2- post-trauma sequelae and services; and 3- long-term impact. Patients described the long-term burden including chronic pain, poor mobility, anxiety or depression, and limited activities of daily living.

**Discussion:**

Civilians injured during the July 2006 war described the traumatising events they endured during the war and the limited access to medical care during and post-war. Up until this study was conducted, affected civilians were still experiencing physical, psychological, and financial sequelae. Acknowledging the limitations of this study, which include a small sample size and recall bias, the findings underscore the necessity for the expansion of services catering to civilians injured during wartime.

## 1 Introduction

The scourge of war and armed conflicts is becoming more prevalent worldwide, more so in low- and middle-income countries (LMICs) (1). Between 2007–2011 and 2012–2016, the average mortality rate attributed to these conflicts more than doubled (2). This escalating instability and prevalence of armed conflicts significantly contribute to not only increased mortality but also heightened morbidity, disability, and diminished quality of life (3). Of particular concern is the growing impact on civilians, especially within the MENA region (4). Families residing in conflict-ridden areas face heightened exposure to a range of hazards, including weaponry, explosions, motor vehicle incidents, structural damage, and mine detonations. These perilous circumstances render families susceptible to a spectrum of injuries, including multiple organ trauma, internal bleeding, traumatic brain damage, penetrating wounds, burns, fractures, amputations, and various ophthalmological and respiratory afflictions (5–7).

Prolonged exposure to warfare and the persistent presence of social, political, and economic instability can give rise to what is termed an “ecology of war.” This concept encapsulates the encompassing environment within which individuals affected by enduring conflicts reside. This environment is characterized by substandard living conditions, limited healthcare accessibility, compromised food and water safety, heightened susceptibility to infections and injuries, impoverishment, widespread human suffering, and even loss of life (8). The ability of civilians to obtain essential care is further impeded due to deliberate targeting of healthcare facilities and vehicles responsible for medical transport (9).

Moreover, the reach of the ecology of war extends to the realm of psychological well-being, with survivors often grappling with an elevated prevalence of conditions such as post-traumatic stress disorder (PTSD), anxiety, and depression (10). Additionally, the enduring repercussions of war, encompassing recurrent hospitalizations stemming from disease, disability, and amputation, compound the already heightened psychological distress experienced by patients (6, 11). The impact on children, while not always readily apparent, is undeniably profound. Children who have endured trauma often exhibit a range of distressing symptoms, including difficulty with bedtime routines, attention-seeking behavior, dependency issues, diminished concentration, persistent worries and fears, and episodes of temper tantrums (12, 13).

In addition to the individual disruption, armed conflicts impose a significant economic strain on both the populace and the government. These conflicts disrupt essential food production and distribution systems, resulting in resource shortages, malnutrition, and the redirection of vital resources away from key economic sectors (14). The resulting substandard living conditions and diminished quality of life compel numerous families to abandon their homes and seek refuge elsewhere, thereby compounding their financial burdens (15).

In the past few decades, Lebanon endured a perpetual political turmoil and several armed conflicts. These include the protracted civil war of 1975–1990, the Syrian occupation of 1976–2005, the Palestine Liberation Organization (PLO) operations in 1960s-1982, and multiple Israeli invasions, attacks, and wars (1978, 1982, 1993, 1996, 2006). As a result, Lebanon suffered from a large burden due to war-related injuries, which represented the highest proportion of injuries in the country (16). Before the civil war, the Lebanese economy thrived; nevertheless, the war had a significant negative impact on the public healthcare system, prompting the government to increasingly rely on the private sector (17). In 1993, the government adopted a health sector strategy aimed at strengthening the Ministry of Public Health’s role (17). In consideration of the health sector reform implemented post the civil war, our focus became on examining the health outcomes of war injuries, particularly in a conflict that had a profound impact on civilians in the aftermath of the civil war. We aimed to conduct a study on the 2006 war in Lebanon, considering it as the most recent devastating conflict endured by the population. This focus is particularly crucial as we explore the long-term impacts of injuries, and selecting an earlier war might have been influenced by the lasting effects of the 2006 war.

In July 2006, Israeli forces waged a 33-day war on Lebanon, hereinafter referred to as the July 2006 war. At least 1,100 individuals were killed and around 4,400 were injured – the vast majority of whom were civilians (18, 19). The war started on July 12th and continued until a ceasefire came into effect on August 14th, causing the destruction of more than 500 sections of road, approximately 100 bridges, and nearly 1,500 buildings, including 30,000 housing units, as well as the internal displacement of nearly 1 million individuals (18, 19). The war weakened all sectors of the country’s economy, significantly impacting its infrastructure, agriculture, tourism, and industrial sectors requiring years of recovery (14, 20, 21). While some studies have addressed specific aspects such as access to maternal care during the war (22), the short-term impact of the war on women (23), the discourses on the effectiveness of pollution legislation during the war (24), the effects of demographic and socio-economic factors on displacement during the war (25), the short and long-term physical (26, 27) and psychological (28) impact of cluster munitions, and the short-term psychological impact of the war (29–32), the collective body of evidence on the topic remains insufficient. This gap extends to areas such as the overall access to healthcare and the long-term outcomes associated with the July 2006 war. Essential questions remain unanswered, including: did patients experience unimpeded access to healthcare services during and post-war? Were they afforded access to essential medications throughout this period? Did patients have access to comprehensive support both in the short and long term? How have war injuries affected them over the long term? Systematically documenting these long-term outcomes serves as a pivotal guide for policymakers, allowing the formulation of evidence-based strategies applicable to global instances of aggression.

This study sought to understand the multidimensional burden of war injuries incurred on civilians during the July 2006 war in Lebanon. We specifically aimed at identifying and evaluating civilians’ access to healthcare and medicine under conditions of war, the long-term socioeconomic burden of injured civilians, and their subsequent quality of life.

## 2 Methods

### 2.1 Study design

We adopted a parallel mixed-method research design, wherein the quantitative and qualitative components were complementary, with a predominant focus on the qualitative aspect. The qualitative data was supplemented with descriptive clinical and financial data collected from hospital medical records alongside a self-rated health questionnaire, the EQ-5D-5L instrument (33). Employed to evaluate participants’ health state at the time of the interview, the EQ-5D-5L questionnaire provided a quantitative overview of the enduring impact of the trauma on their long-term well-being. Conversely, the qualitative component facilitated a nuanced exploration of patients’ experiences on the day of injury and thereafter, providing a comprehensive understanding of the circumstances during and after the war, particularly in the long term. While identifying an unmet need may be straightforward, grasping the intricacies of war patients’ experiences necessitates a profound examination, achievable through qualitative analysis. Given that participants’ injuries occurred over a decade prior to the start of this study, we gathered clinical and financial data from medical records to mitigate the risk of recall bias. Although the quantitative aspect predominantly aimed to address recall bias, the qualitative research assumed a central role in shaping the overarching themes and subthemes of the study. The study involved two hospital sites (A and B), one of which is a regional centre for trauma care situated in Beirut, and the second is a large hospital in the Beqaa area, which was at the forefront of the battlefield. The study was conducted in accordance with the Declaration of Helsinki, and the ethical approval was secured from the Institutional Review Board (IRB) at the American University of Beirut (SBS-2019-0226).

### 2.2 Recruitment and sampling

We used a purposive sampling approach to identify and recruit consecutive patients admitted to the two hospitals. Patients who sustained war-related injuries during the July 2006 war in Lebanon were included in our study. Patients who died as a result of the trauma or who were lost to follow-up were excluded from the study.

The two hospitals in our study varied in their systems, impacting how eligible patients were identified. Hospital A halted elective surgeries during war or disaster, focusing on emergency and life-saving procedures. This protocol, active during the July war, facilitated the identification of patients with war injuries. Conversely, Hospital B used an electronic filter, enabling the identification of those injured in the July 2006 war. Staff at Hospital B retrieved data through retrospective reviews of admitted patients’ records.

Patients identified from medical records, with available contact details, were initially approached by their healthcare providers for preliminary approval to be contacted by our research team. Due to the COVID-19 pandemic, recruitment and data collection procedures were adapted as follows:

- Pre-COVID-19 Recruitment: after securing patients’ consent, we telephonically briefed them about the study and invited them to a face-to-face interview at a mutually agreed time and place. Older adult participants aged 64 and above underwent cognitive assessment using the validated Arabic version of the General Practitioner Assessment of Cognition (GPCOG) tool (34). Scores between 0 and 4, indicating possible cognitive impairment, led to exclusion. Interviews were recorded with consent, and participants completed the EQ-5D-5L questionnaire.
- COVID-19 Recruitment: in-person interviews shifted to phone interviews. The Abbreviated Mental Test (AMT) replaced GPCOG, with scores below 6 suggesting possible cognitive impairment (35). AMT was adapted to Arabic, monitored by an expert. Interviews were conducted privately, recorded with permission, and consent forms were shared via WhatsApp or email. The EQ-5D-5L questionnaire was read by the researcher, and responses were documented.
- All recordings were securely stored on a password-protected computer.

Among the 245 eligible patients identified from hospital records, 162 could not be reached due to invalid or missing phone numbers. Additionally, 10 patients passed away after the war, and 26 did not respond to initial and follow-up calls. Of the remaining 47 patients, 22 declined participation, resulting in a final sample size of 25 participants.

### 2.3 Data collection

#### 2.3.1 Quantitative data collection

A retrospective medical records review was conducted to retrieve data on patients admitted to hospitals A and B due to the July 2006 war. Costs of procedures and surgeries conducted on the identified patients were retrieved from the patient billing department at Hospital A and from patient records at Hospital B. Data collected from one or both hospitals, depending on availability, included patient number, age, gender, date of injury, date of admission, date of discharge, place of residence, hospital transfer information, past medical history, previous physical disability, type of injury, injury mechanism, location of injury, amputation, type of procedures, and short and long term complications.

The EQ-5D-5L self-complete paper version questionnaire consists of two components, the EQ-5D-5L descriptive system and the EQ Visual Analog scale (EQ VAS). The descriptive system comprises five dimensions (mobility, self-care, usual activities, pain/discomfort, anxiety/depression). Each dimension has 5 levels: no problems, slight problems, moderate problems, severe problems, and extreme problems. Due to the small sample size in our study, the 5 levels were divided into two categories to avoid small cell size reporting, no problems (level 1), and problems (levels 2–5). The EQ VAS records the respondent’s self-rated health on a 20 cm vertical visual analogue scale with endpoints labeled ‘the best health you can imagine’ and ‘the worst health you can imagine’. The questionnaires in Arabic and English were obtained following registration of the study through euroquol under ID: 30253.

#### 2.3.2 Qualitative data collection

The researcher (EK) received training on the ethical and responsible conduct of research and conducted a mock interview with another member of the research team prior to data collection. EK had a brief discussion with participants prior to the start of the interviews to introduce herself, her work at the Global Health Institute, and the aims of the project. EK conducted a total of 25 semi-structured interviews, which was deemed sufficient to reach saturation in our qualitative analysis. The interviewed patients recounted a spectrum of experiences ranging from relatively mild to severe. This breadth allowed us to obtain a comprehensive understanding of the diverse experiences encountered by war-injured individuals. Consequently, further recruitment of patients was deemed unnecessary, as the primary objective of our study was to comprehend the nuanced burden on patients rather than quantifying it. Additionally, given the widespread sheltering of people during the war, our participants’ experiences are representative of a broader population within their surroundings. Twelve face-to-face interviews and 13 phone interviews were conducted between August 2019 and June 2020, across Lebanon. Interviews were recorded when permitted to do so by the participant (*n* = 22, 88%), otherwise, detailed notes of the interviews were taken (*n* = 3, 12%). All interviews were conducted in Lebanese Arabic, a dialect of Levantine Arabic mostly used in Lebanon, and all participants agreed to be quoted in an anonymous form. Using a topic guide (Supplementary material S1), questions addressed the description of the day of the incident, participants’ access to services and support, the financial and non-financial burden caused by the injury, and the long-term impact of the injury on the participants as well as on their caregivers and dependents. To ensure clarity and relevance of questions, we consulted experts in the fields of qualitative research and medicine, specifically war-related trauma, for the development of the topic guide. The interviews lasted approximately 20 min on average. Interviews were transcribed in Arabic and relevant quotes were translated into English. All transcripts were anonymised using a unique identifier for each participant. The assigned code, used for citation purposes below, was composed of a letter that refers to the hospital the patient was admitted to, followed by a number that represents the chronological order of the interview. For example, A4 codes for the 4th interviewed patient; that patient was admitted to hospital A.

### 2.4 Data analysis

#### 2.4.1 Quantitative data collection

We used Excel to analyse and explore simple descriptive statistics on the frequency and percentage distribution of demographic, injury, cost, and questionnaire data. Simple statistics were also used to analyse socio-economic data collected from interviews, such as the duration of absence from work, out-of-pocket expenses paid for post-war treatments, and patients who changed or quit their jobs due to disabilities.

#### 2.4.2 Qualitative data collection

We used NVivo 12® for data management and thematic analysis. We adopted the 6-phase process described by Braun and Clarke (36) to thematically analyse the data. In phase 1, members of the research team read and re-read transcripts to get acquainted with the information. In phase 2, descriptive coding line-by-line for each transcript was conducted. In phase 3, an initial coding framework was generated by two team members (EK and NE) which was then reviewed by an expert in qualitative research (GHA). In phase 4, the research team discussed the relationships between codes. A log of potential themes and sub-themes was developed, including a list of definitions and quotes to illustrate each theme and sub-theme. In phase 5, the list of candidate themes and sub-themes was further refined based on consensus among the research team members involved and to highlight the existing relationships between them. As for phase 6, the findings were presented in a narrative form, and a synthesis of the results was included. These findings were supported by quotes from interviewees relating to identified themes and sub-themes.

Findings were reported based on Good Reporting of A Mixed Methods Study (GRAMMS) criteria (37).

### 2.5 Integration of findings

Integration occurred at two levels, at the methods level and at the interpretation and reporting level. Integration through methods occurred through ‘connecting’ as both types of data collected from interviews, medical records, and self-rated questionnaires link through the sampling frame. Integration of data at the interpretation and reporting level occurred through the ‘narrative’ and ‘data transformation’ approaches. Findings were presented in a single report, although some of the quantitative data were reported separately and others were converted and integrated into the qualitative data.

### 2.6 Increasing rigor

To increase rigor, we focused on demonstrating both credibility and reflexivity. Regarding credibility, all discussions, except 3, were audio-recorded, transcribed verbatim, accurately translated into English whenever necessary, and utilized as the main data repository. As for reflexivity, and to limit biases, most team members were involved in the analysis and interpretation of the results. As for confirmability, we used a semi-structured interview approach to guide the discussion to improve consistency across interviews. The integration of quantitative and qualitative data increased the confirmability and dependability of the findings.

## 3 Results

### 3.1 Demographic data

Eight participants were recruited from hospital A, and 17 from hospital B. The sample was composed of 25 participants, predominantly male (68%), with a mean age of 27 years at the time of injury (SD = 8.4). According to medical records and interviews, the marital status was ‘single’ at the time of injury for more than half of participants (52%; *n* = 13) and ‘married’ or ‘engaged’ for 44% (*n* = 11). Marital status was undetermined for one of the participants at the time of data collection. Most participants reported being injured in Baalbek governorate (68%; *n* = 17), followed by South Lebanon (20%; *n* = 5) and the capital, Beirut (12%; *n* = 3).

Blast-related body shrapnel, burns, and wounds comprised the highest proportion of injuries (84%; *n* = 21). The other 16% (*n* = 4) of injuries were caused by blast-related falls. Six out of 18 patients, who underwent operational procedures according to their medical records, required complex wound repairs including debridement, 4 of which were transfer patients. According to the medical records relating to their first admission, patients underwent 3.2 operational procedures on average (Table 1). When answers from interviews were considered, most patients were found to have undergone multiple surgeries as well as revision surgeries with a mean total of 7 procedures ranging between 1 and 60.

**Table 1:** Clinical findings based on medical records.

	Hospital A	Hospital B	Average
Type of injury (decreasing order)	Penetrating wounds (puncture wounds, burns) Blunt force trauma [lesions (lacerations, abrasions, and contusions), fractures]		
Average length of stay (number of days)	14.6	1.5	6
Average number of procedures per patient	4	2.8	3.2

Participants were not fully functional until, on average, 9 months post-injury. Four participants had to change or quit their jobs due to disabilities caused by their war-related injuries. Treatment of those injuries cost on average 5 million Lebanese Lira (L.L.) (3,333 USD), which were covered by the Ministry of Health. An additional average of 3.5 million L.L. (2,333 USD), ranging between zero and 30 million L.L. (20,000 USD), were covered by patients post-war for the treatment of complications (Table 2).

**Table 2:** Socio-economic findings based on either medical records or interviews.

	Hospital A	Hospital B	Average
Average duration until patients became functional post-injury (months)	14	5	9
Average total healthcare costs paid by the Ministry of Health for war-related surgeries and treatments per person for the duration of their stay^a^ (L.L.)	11,991,582 (7,994 USD^b^)	1,878,792 (1,253 USD)	5,114,884 (3,410 USD)
Average total out-of-pocket expenses paid for post-war treatments per person (L.L.)	6,386,250 (4,258 USD)	650,000 (433 USD)	3,349,412 (2,233 USD)
Proportion of patients who changed or quit their jobs due to disabilities (%)	8	8	16

L.L., Lebanese Lira.

USD, United States dollar.

^a^Based on medical records.

^b^Based on the exchange rate at the time of the war: 1 USD = 1,500 L.L.

**Figure 1:**
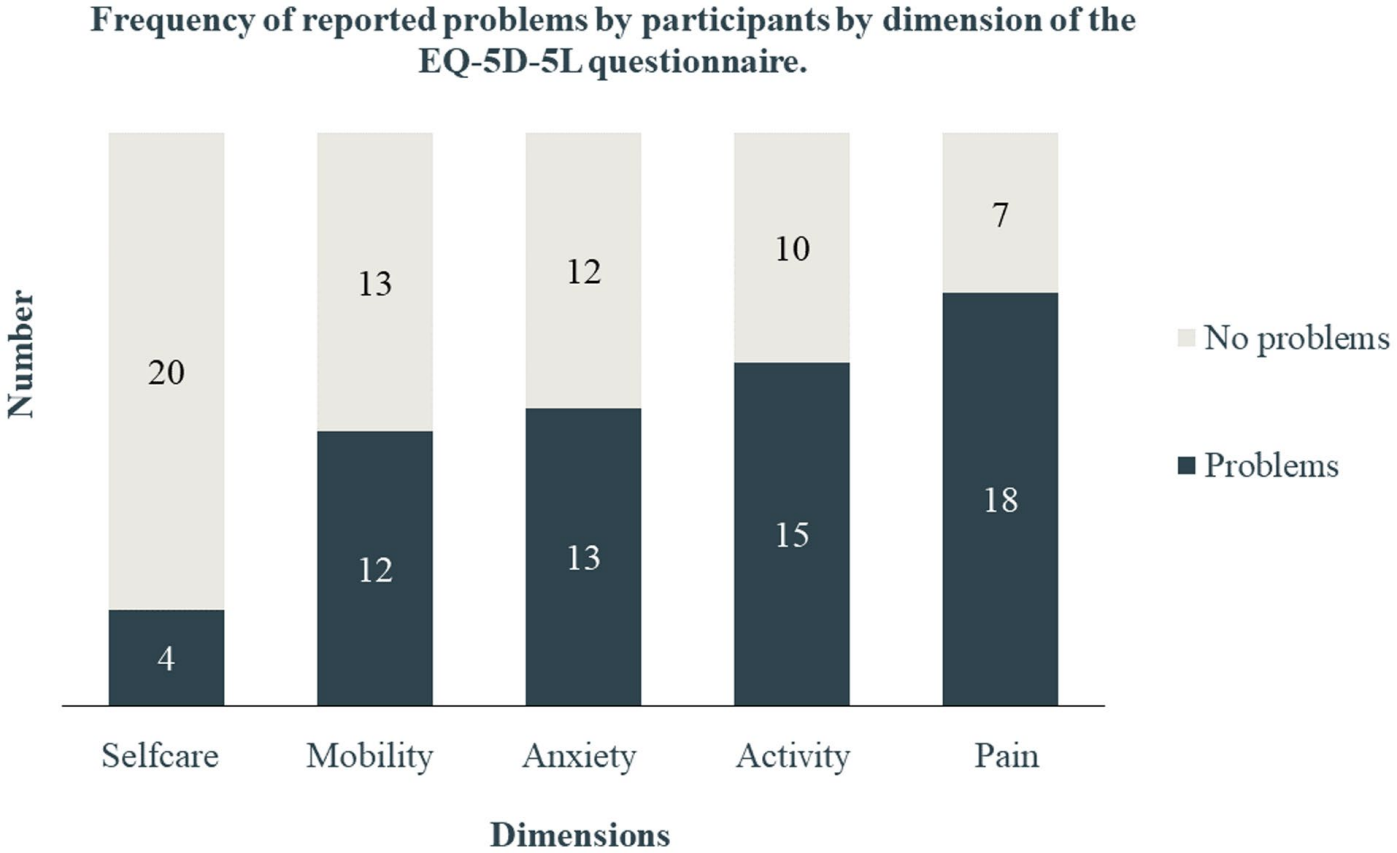
Frequency of reported problems by participants by dimensions of the EQ-5D-5L questionnaire.

The mean EQ VAS was 60 (SD = 26), slightly higher in males (61, SD = 25) than in females (57, SD = 32). Participants reported problems at all dimensions of the EQ-5D-5L instrument, more so in pain (72%), usual activity (60%), anxiety (52%), and mobility (48%) (Figure 1).

### 3.2 Emerging themes

The thematic analysis from the interviews revealed three main themes that describe patients’ experience chronologically, from the day of the incident until the day of the interview: 1- recall of the time of the incident, the thousand miles journey, and patients’ access to services, 2- post-trauma sequelae and services, and 3- long-term impact. Within each theme, we identified several sub-themes (Table 3).

**Table 3:** Summary of the main themes and their sub-themes.

Themes and subthemes	Descriptions
1. Recall of the time of incident, the thousand miles journey, and patients’ access to services	
The mayhem the day the airstrike happened	Description of the missile attacks that caused the injuries.
*“And the thousand miles journey began”*	Description of the referral process from the injury site to the hospital.
Provision of services under exceptional circumstances	Description of the types of services received at secondary and tertiary health centres directly post-injury. Description of and opinion on the governmental response.
Coverage of services	Description of the payment process for the acute treatment of injuries.
2. Post-trauma sequelae and services	
Impact of poor healthcare service quality	Narration of the short-term burden caused by unmet needs regarding the services provided.
Coverage of services	Description of the payment process for the treatment of readmissions.
Impact of disabilities on patients post-trauma	Narration of the short-term burden caused by disabilities post-injury.
Role of caregivers	Narration of the role of family members, friends, and caregivers post-injury.
3. Long-term impact	
*“She had to quit to care for me”*	Narration of the long-term impact of the participants’ injuries on their caregivers.
The war, *“engraved like a seal”* in their minds	Narration of the long-term physical, psychological, and financial burden of the war injuries on participants.

#### 3.2.1 Theme 1. Recall of the time of the incident, the thousand miles journey, and patients’ access to services

When asked about the day of the injury, participants thoroughly described the sequence of events from the start of the day until the time of injury, their transfer to the hospital, and the types of services they received. Participants also often provided a background to the story to explain their location at the time of the injury.

##### 3.2.1.1 Subtheme 1. The mayhem the day the airstrike happened

All interviewees vividly described the situation the day they were hit by the missile. Many indicated that when people were informed about an imminent attack, they evacuated their towns. Yet, their evacuation routes were targeted by airstrikes:

*We were all leaving as a convoy… All of us meaning there were about 1500 people. The cars moving, leaving* […] *and I got hit, my mother too, and my brother-in-law died. But not from the same missile. Each one* (was hit by a different missile) (**A7**)

Others witnessed the attacks right where they live. They saw their homes or that of their neighbors destroyed in a split of second, where families had gathered seeking shelter: *“we were around… 30 people”* (**A10**).

They remembered “walls collapsing,” “shattered glass all over the place,” “bodies burning,” being “projected in the air,” and “rising from the rubble” with blood covering their faces and bodies. Some even witnessed the death of family members and neighbors.

During the attacks, conditions became intense and chaotic, leaving injured civilians, especially children, in a state of confusion and terror. One participant, who was a child at the time, described his ‘illogical’ behaviour following a blast, caused by his panic: *“In a state of mayhem, I did not know what to do, and how to act, I started running in the street”* (**A6**).

Some patients only remember the missile attack, after which they lost consciousness or memory of the events leading to their hospitalization: *“I suddenly realised that I was in the hospital”* (**B16**).

On these highly kinetic military action days, several participants recalled that their transfers to hospitals got delayed, either intentionally or involuntarily, until the situation had settled down. One participant, whose leg was injured, had to wait all night near the location of the incident for the situation to abate for him to be found and transported to a hospital.

*When I got injured, I got injured at night […] but it wasn’t until the morning that I was transferred to the hospital because of aircrafts […]no one dared to go out […] I stayed sitting in a nearby area* (**B24**).

##### 3.2.1.2 Subtheme 2. “And then the thousand miles journey began”

Participants went on to describe the referral process from injury sites to hospitals. The primary first responders were the Lebanese Red Cross (LRC), only in the areas where no restrictions were imposed by Israeli forces.

Faith-based non-governmental organizations (NGOs) and community members were also involved in the search and rescue of injured patients.

One participant said he had to drive himself to the nearest ambulance while his hands and body were burning:

*We got burnt and our skin melted,* […] *I was looking at myself and my skin was peeling, and my condition was very bad. But we didn’t lose consciousness, we got up and quickly turned on the car and went to Rmeich. Yes, we went by ourselves, the Red Cross is forbidden from passing by* […]*. Yes, at that time they couldn’t. We, ourselves, turned on the car and went to Rmeich, to the Red Cross; and the Red Cross… truthfully, we thank it a lot, it did its job, and transferred us to Tyre, as a first step, and the long journey began, from Tyre to Beirut* (**A5**).

To complicate the matter, rescue teams and some hospitals were attacked and targeted by airstrikes putting healthcare professionals in danger and further hindering the transport of patients to healthcare facilities.

The transfer to hospitals was, to say the least, an exhausting journey *“For the person in pain, time has value,” “I arrived at* […] (the referral hospital)*, collapsing, to be honest, worn out and my wound starting to hurt”* (**A5**). The roads to the hospitals were treacherous. Many bridges were bombed limiting access to the capital or metropolitan cities, where leading hospitals were situated. Although dangerous, several participants were transferred from one city to the other because hospitals were either unequipped to treat serious injuries or were raided leading to the evacuation of those hospitals. Ten participants reported being transferred from one health facility to another, four of whom had to endure three or more transfers.

##### 3.2.1.3 Subtheme 3. Provision of services under exceptional circumstances

Given the exceptional circumstances when the injuries were inflicted, it was not surprising that most participants pointed to several unmet needs.

Several participants pointed out that initial debridement was suboptimal: *“It got infected* […] *they had not cleaned before stitching. You know? They noticed that something was removed, they stitched. And they stitched incorrectly”* (**A7**).

Several patients had shrapnel retained in their bodies, which they were unaware of after initial hospitalization. Eventually, they necessitated readmissions, which could not always be concurred by secondary data because participants resorted to different hospitals: *“I travelled to Saudi Arabia* […] *there, they completed the treatment”* (**A3**). In some cases, the reported inadequate treatment led to chronic pain: *“he did not clean* (the wound) […] *he stitched…* […] *he directly stitched and there were… pebbles in my arm…* […] *I started feeling pain because of it.”* (**B9**).

Given the urgency of the matter, many participants received emergency services in community hospitals, that often were missing on essential services and equipment, before being transferred to specialized hospitals.

The chaotic environment triggered by the war might have led certain healthcare professionals to take ill-conceived decisions. One participant described how she successfully eluded the avoidable amputation of her arm, which was fully functional at the time of the interview.

*They wanted to amputate my hand and my husband came and saw what was going on* […] *he didn’t, he didn’t let them amputate it* […] *Thank God, I can move it now, as normal* (**A17**)

Various remarks from participants alluded to a lack of a robust emergency preparedness plan, whether by national authorities or by hospitals. Some participants recognized the gravity of the situation and the necessity for medical triage in the chaotic environment they were in: *“At the time, the emergency room was very crowded and… my case was nothing compared to the other cases I witnessed there, in the emergency room… burned victims, victims with amputated legs, victims with amputated arms”* (**A6**). However, few participants complained about the delay in their treatment which prolonged their suffering and increased their risk for complications: *“They kept us in a room and did not check on us until the next day or until the middle of the night…”* (**A7**).

In addition to the lack of coordination in hospitals, the inadequate support expected from national authorities was also noted. Following the high influx of internal displacement, participants reported that no official national plan was implemented to provide refuge to those in need.

*“When there is an evacuation this way, and in these… large numbers, people go and they resort to schools, to… they go to churches, to… they go to hospitals* […] *people migrate and they leave, and eventually when they leave the danger zone which is the essential thing, they start thinking where to go. Maybe, God forbid, the government considers setting up a safe compound, a camp… for people to resort to”* (**A5**)

##### 3.2.1.4 Subtheme 4. Coverage of services

The costs of hospitalizations during the month of the war, including readmissions, were covered by the Ministry of Health. The average cost of hospitalizations for hospital A was around 8,000 USD, 1,250 USD for hospital B, and 3,500 USD for both.

Table 4 summarises more specific quotes related to theme 1 and its subthemes.

**Table 4:** Theme 1 – list of sub-themes and supportive quotes.

Subthemes	Supportive quotes
1. The mayhem the day the airstrike happened	
Description of missile attacks	*“I told him that the aircraft became too low, the MK became low, let us leave before it hits us. I did not finish the word and it hit us”* (**A5**)
	*“So, they are a form of missiles, a form of a comb* (joint fires) *meaning they were combing* (striking missiles at) *any movement they would see”* (**B24**)
Narration of incident	*“And I stood up after regaining consciousness, I walked but I did not know where to because I could not see anymore and I could not hear anymore, I lost almost all kinds of sensation, but I was walking…* “(**A3**)
	*“My sisters and mother died because of the collapse that happened on them, or rather the quantity of concrete”* (**B8**)
	*“The rocks mostly hit my back and my head, but then my head started to swell, and blood started to quickly appear on my face, I could not see anymore”* (**B18**)
Description of the destruction of homes	*“The entire house collapsed on us”* (**B10**)
	*“I started hearing walls falling* […]*”* (**B13**)
	*“The aircrafts hit the house that is directly next to my brother’s house, meaning the neighbours* […] *They died, they all became martyrs”* (**B15**)
Description of the chaos	*“I looked at their faces, I do not know if you noticed those children in Qana centre, do you remember them?* (referring to the Qana Massacre in a village in Southern Lebanon in 1996) […] *How their faces look burnt and like that* […] *Yes, this was how my children looked like”* (**A17**)
2. *“And then the thousand miles journey began”*	
Identification of referral processes: Lebanese Red Cross Faith-based NGOs Community members Description of the delay in transfer due to Restriction of movement Attacks on medical teams and hospitals	*“I wanted to stop a van or a taxi or a car to take me to the hospital* […] *an autocar comes accelerating, wanting to go towards the location of the blast, our neighbor, may God bless him”* (**B13**)
	*“The Risala Scout came to take us, they got hit by a missile, three personnel from the Risala Scout martyred”* (**A1**)
	*“And I could not go to the hospital that day, the streets were also patrolled, every car, everything that came out…, I waited until the next day while suffering from the pain”* (**B12**)
	*“On that night* […] *it was not possible for anyone to go to the hospital, I had to wait until the next day to go”* (**B15**)
	*“I waited for not more than half an hour until someone came, but they waited, in case… they were afraid of a second attack”* (**B17**)
	*“I started bleeding, and I could not stand on my feet anymore and it stayed like this almost until the morning just until the situation calmed down”* (**B25**)
3. Provision of services under exceptional circumstances	
Unmet need – suboptimal debridement	*“The doctor saw me and said that my case cannot be treated here,” “I have a shrapnel here, it was inside, I had told them that there was pain here, they tell me it is just caused by the impact of the blow* […] *It turned out to be a shrapnel on my lower jaw, that they did not detect, they removed it* (at the 3rd hospital)*, thank God I can move it now, at first I could not move my jaw. They also removed a few shrapnel from my eyes and arm”* (**A3**)
	*“They only sutured my face, and probably for that reason, a lot of shrapnel remained in the wound, and I was retreated* (at another hospital)*”* (**A7**)
	*“She used to do physiotherapy for me which hurt me due to the rocks and pebbles that were in my arm. At the end I resorted to* (another hospital)*”* (**B9**)
Unmet need – unequipped hospitals	*“They sutured us there* (at the first hospital) *without anaesthesia, the stitches still show* […] *then* (at the second hospital)*, they removed the stitches and sutured us all over again and put us under anaesthesia”* (**A1**)
4. Coverage of services	

#### 3.2.2 Theme 2. Post-trauma sequelae and services

The short-term sequelae of the trauma and the impact of the quality of services were substantial and included consequences that increased patients’ physical, psychological, and financial burden.

##### 3.2.2.1 Subtheme 1. Impact of poor healthcare service quality

At times, mismanagement of some injuries led to short and long-term complications: *“15 days passed by… I got an infection”* **(A3)**. According to medical records, these complications included infections caused by multi-drug resistant organisms. Many participants were readmitted to hospitals for repeat surgeries or to treat complications potentially caused by inadequate initial procedures. Post initial hospitalization, one participant underwent more than 50 surgeries to treat a supposed complication caused by an error at initial hospitalization.

Healthcare services seemed to be mainly focused on tending to the physical needs of injured civilians, neglecting their mental health, and possibly worsening it. One participant, who was a child at the time of injury, described his traumatising and stressful experience at the hospital which was aggravated by a healthcare personnel’s behaviour.

*I was mostly scared of… them amputating* (my hand) *[…] at the time… I kept asking the doctor, will you amputate it? He said no, then I insisted that they will amputate it, in the end when he got annoyed by my repeated questions, he said: enough, yes, they will amputate it… […] At that point, I started to feel stressed* (**A6**).

##### 3.2.2.2 Subtheme 2. Coverage of services

The Ministry of Health covered the cost of hospitalizations during the war, but not beyond the one-month war. Therefore, many of these readmissions were either paid by insurance companies or out-of-pocket. In addition, faith-based organizations also provided financial and medical aid to certain civilians.

Some participants resorted to physicians and therapists who performed *pro bono* procedures and physiotherapy sessions. Other participants benefited from donations offered by national and international funders, including medical services and devices such as hearing aids. However, those opportunities were limited, and many patients were unaware of their availability.

NGOs were also highly present during this war, providing aid to healthcare facilities and civilians, including prosthetics.

##### 3.2.2.3 Subtheme 3. Post-trauma implications of disabilities on patients

Disabilities following war injuries impacted most of patients’ daily living in the short run. For months post-injury, many patients were unable to perform basic activities of daily living and were therefore dependent on others.

Few participants struggled with injury-specific difficulties. Several participants had difficulty driving, one of whom was a taxi driver whose work was substantially affected by his disability. One participant with an upper limb amputation explained how wearing a donated prosthesis was uncomfortable, in which case he preferred not to wear the device *“I did not adapt to it* (the prosthesis) *at all, it bothered me more than it relieved me”* (**B19**).

Another participant with facial and upper body burns suffered from sun sensitivity for a while post-conflict *“I used to hold an umbrella, because I could not handle the heat of the sun in the beginning.”* (**A5**).

Not being able to care for themselves and being dependent on others post-injury caused two female participants to feel like a burden.

*There is someone helping you, and you have young children, this is when you feel like a burden, a burden, you understand me? And you know in days of war no one tolerates anyone, not even themselves […] Yes, this is what affected me* (**B12**).

##### 3.2.2.4 Subtheme 4. Role of caregivers

Due to their conditions, most patients were dependent on others for a certain period. Most participants were cared for by family members, mainly mothers and sisters. This was often the case because most mothers and daughters were housewives who had time to tend to the participants’ needs. Engaged/married male patients were also cared for by their fiancés/wives. One participant was however able to hire a nurse whose services were covered by his insurance.

Table 5 summarises more specific quotes related to theme 2 and its subthemes.

**Table 5:** Theme 2 – list of sub-themes and supportive quotes.

Subthemes	Supportive quotes
1. Impact of poor healthcare service quality	
	*“it all got infected* […] *they did not clean* (the wound) *well when they were suturing, you know? They noticed that something was removed, they sutured. And they sutured incorrectly* […] *two shrapnel were left, during my second operation at* (the referral hospital)*, they opened and found two shrapnel”* (**A7**)
2. Coverage of services	
	*“They said in the ministry that they will stop paying for my treatment, my chart was closed… Because the war had ended”* (**A3**)
3. Impact of disabilities on patients post-trauma	
	*“For some time, I could not use my hands for anything… When I left the hospital, I stayed around a month and a half requiring the help of my wife… helping me, undressing me, dressing me. Even the food, the situation was bad while eating”* (**A5**)
4. Role of caregivers	
	*“I was engaged not yet married, yes, she used to care for me* […] *until she moved in* […] *between my injury and my wedding, there was maximum 15 days* […] *she was a nurse too […] she used to care for me* […] *until I adapted to my situation”* (**B19**)

#### 3.2.3 Theme 3. Long-term impact

Injured civilians’ suffering and the burden on their caregivers did not end after a few months or one year, but rather lasted more than 10 years. Participants described their agony throughout those years on the physical, physiological, and financial levels.

##### 3.2.3.1 Subtheme 1. *“She had to quit to care for me”*

Few caregivers had quit their jobs to nurse the patients back to health. They looked after them until they became functional again and experienced no issues with self-care.

Caregivers were reported to be understanding and supportive. One participant reported that his wife had quit her job to care for him and their children, after which she had to work again to provide for the family.

*after my injury she stopped working […] and there were children […] and I can’t* (manage) *on my own at the house, I mean children and whatsoever […] next to our house there’s a school where my uncle and aunt work […]* (my wife) *got hired as a school nurse at that school only so that we are able to […]* (survive) (**B19**).

##### 3.2.3.2 Subtheme 2. The war, *“engraved like a seal”* in their minds

###### 3.2.3.2.1 Subtheme 2.1. Physical burden

War injuries had long-term implications on patients’ physical health. Participants experienced chronic pain, difficulties with day-to-day activities, and temporary or permanent disabilities.

Ten participants reported chronic pain which still prevents them from carrying out certain activities, such as walking long distances, playing certain sports, or carrying groceries. One participant suffers from excruciating pain whenever she sleeps on her injured arm, two participants endure painful winters due to colder weather, and one patient experiences acute neuropathic pain when his injured hand collides with hard surfaces.

Other participants still endure daily struggles due to their disabilities, such as unilateral hearing loss and amputated fingers or arms. One participant, whose shoulder got dislocated, explained how he must be cautious while carrying out everyday activities to avoid re-dislocations.

*It always kept happening to me […] every time it dislocated, I went back to the hospital. […] It kept on getting dislocated, all the time. […] any wrong movement, any wrong load […] even when* (I) *[…] stretch […] there is a chance that my shoulder would dislocate* (**B14**).

###### 3.2.3.2.2 Subtheme 2.2. Psychological burden

As some participants have pointed out, war affects most people of all ages *“the war […] it affects the young and the old, not to lie to you, it affected a lot* (of people)*, everyone […] not just me I mean”* (**B20**). These patients not only complained about the physical trauma of war but also reported negative psychological symptoms which have been affecting their health ever since the war. People witnessed unimaginable atrocities which created painful memories that return to torment them decades post-war: *“But at that time… at that time, I used to see things that I have never seen in my life”* (**A6**).

Witnessing the aftermath of the war, their destroyed cities, towns, neighborhoods, and homes, filled victims with a sense of sorrow: *“*(It looked like) *a place where an earthquake happened, a destructive tornado… there is no house, there are no, there are no human beings… something, something very miserable, truly very horrifying”* (**A5**).

Others described the sound of missiles which keeps on haunting their minds: *“until now […] whenever I hear about war, or hear about an aircraft, […] hearing its sound, I get annoyed and stressed […] Whenever I hear the sound of aircraft or something, I get a headache”* (**B21**).

Some participants are tormented by their own thoughts and have developed chronic anxiety due to their traumatic experience: *“A large burden […] I cried a lot; you could not believe how much, like I barely started talking to you and started crying”* (**B20**).

Many participants reported developing mental illnesses, including anxiety and depression. To help with their miseries, few participants admitted to taking antidepressants or anti-anxiety drugs. Following the loss of her unborn child due to a blast from the July 2006 war, one participant became depressed and socially isolated, affecting her as well as those around her: *“I would not want to see anyone anymore […] just sit on my own”* (**B25**).

These war injuries and their resulting disabilities have impacted patients’ quality of life. Few participants reported having feelings of emptiness after losing their jobs due to their disabilities. These individuals did manual labor before the incident and have therefore lost the opportunity to contribute to their family and society, leaving them feeling useless.

Some participants lost the ability to conduct certain daily activities, and therefore the ability to adhere to traditional gender roles. It was mainly the male patients’ gender role conflict that affected their psychological state. One patient was ashamed of not being able to undertake his duties as the “man of the house,” such as carrying heavy groceries to his home. Another participant was anguished about not being able to provide for his family due to his injury-related disability, which cost him his employment.

Few patients went through other domestic problems due to their injuries, including fear and disgust by their family members toward their injuries. One participant described the fear in his daughter’s eyes when she saw his injuries, after which she ran away from him. Another participant reported that her husband at the time would ask her to sleep on her painful injured arm to avoid seeing it.

Due to taboos associated with disabilities in Lebanon, few participants have experienced social anxiety. The daughter of one of the participants, who was also affected by the war, developed ocular problems, including strabismus, which she was bullied for by her schoolmates. The bullying of the child affected her and her mother, the participant in our study, whose main concern is her daughter’s physical and mental health: *“Just for my daughter, not more, I am upset for her. […] She has many problems now […] I get really upset for her”* (**B20**).

One participant with an upper limb amputation explained how he withdrew from society after the injury and could not reintegrate directly post-conflict due to the lack of social acceptance. More than a decade post-war and despite his efforts, the participant still describes his social life as “acceptable”: *“I gained courage bit by bit; in principle, socially* (my life) *is acceptable”* (**B19**).

In addition, disabilities might affect parenting techniques as injured adults start appreciating the activities that they can no longer undertake. Following his disability, one participant became more lenient with his son, allowing him more time to play sports instead of spending most of his time studying.

Participants were also victims of losing family members due to the war. Although severely physically injured, their main burden was the loss of their loved ones. Following the unfortunate events, few participants have developed feelings of guilt either for surviving the attacks, contrary to their family members who passed away, or for not being able to provide aid to those who were dying.

The war was not only agonising for adults, but also for children who became orphans in addition to being physically injured. One participant, who was a child at the time of the war, described the difficulties he faced post-war, after becoming maternally orphaned.

*I go study on my own, sleep on my own, my father comes home at the end of the, at the end of the day, yes, at that time […] I would be crying the whole time […] I just want to strangle myself, I want to die” “it wasn’t until I got married until I felt… I understood what attention means in the form of affection* (**B8**).

Although the participant lacked support from his father after the death of his mother, he understood that his father was also suffering and grieving.

*My father is one of those persons whose mental state was severely damaged, his daughters and his mother and his father and his wife died, yes, my father got to the point where he started smoking a carton of cigarettes just to console himself. My father no longer drank or ate, living on cigarettes and coffee […] He got hysterical hysterical hysterical hysterical, yes, my father’s mental state got destroyed* (**B8**).

The participant also complained of a lack of social support in schools, where adults pitied him instead of helping him cope and adapt to this situation.

###### 3.2.3.2.3 Subtheme 2.3. Financial burden

In addition to the physical and psychological burden, injured civilians were affected financially.

On average, it took participants 9 months to regain functionality. Due to this prolonged rehabilitation period and the partial or absent compensation from work, participants’ employment status were affected: *“within 6 months they brought someone to replace me”* (**A1**).

Four out of 19 participants who discussed the topic had to change or quit their jobs due to their war-related disabilities. One participant returned to her work before her wounds completely healed, to keep her employment-based health insurance: *“I was forced to go back to teaching for the NSSF to keep on working […] dismissing my pain and suffering and went* (to work)*”* (**A7**).

Although the Lebanese government covered hospitalization costs during the one-month war, participants without healthcare insurance coverage had to pay for readmissions that occurred beyond the end of the war. Two participants explained how they are still going to their dentists, on a gradual basis to manage the expenses, to get implants following mandibular fracture injuries caused by blasts.

Injured civilians had to pay additional out-of-pocket costs, such as the costs of certain medications and physiotherapy sessions when they did not have insurance or when the latter did not cover them. This was however not possible for all patients as many of them could not afford treatments and rehabilitation services: *“I no longer used it* (the treatment) *because it costs a lot”* (**A7**).

Table 6 summarises more specific quotes related to theme 3 and its subthemes.

**Table 6:** Theme 3 – list of sub-themes and supportive quotes.

Subthemes	Supportive quotes
1. *“She had to quit to care for me”*	
2. The war, *“engraved like a seal”* in their minds	
2.1. Physical burden	
	*“mostly in the winter I… in the winter I cannot do anything… because the fractured fused bone, meaning from my chest to my leg, my arm, they all hurt in the winter… no matter how much I keep them warm they still hurt, only in the winter, in the summer I live a normal life”* (**A1**)
	*“If I slept on it by mistake,* (I) *wake up in the morning in pain,” “I would not let anyone touch me.”* (**B9**)
2.2. Psychological burden	
Pain from losing one’s home	*“Scenes like a nightmare, but like, these scenes do not not not… no matter what happens with the person, and hopefully good days come to everyone, but this scene cannot be removed from* (minds) *and it cannot… it cannot be erased, no! And if God gives us years to live, we will continue to narrate it and talk about it to our children and grandchildren, this suffering… and we hope that the current and future generations do not suffer from similar circumstances…”* (**A5**)
	*“My house was all destroyed* […] *that might have been more difficult for me than* (physical) *pain”* (**A17**)
Chronic anxiety	*“The ringing sound in my ears is always buzzing it does not go away… the sound of the missile* […] *mostly when I want to sleep, the sound becomes louder”* (**A3**)
	*“Sometimes when I remember this… I get… a mental breakdown… I lose my nerves…”* (**A3**)
Feelings of uselessness	*“Disability, it ru… it ruined us… We could not, we could not work anymore. My siblings work in agriculture. I could not work with them anymore, and I could not,* […] *I could not do anything.”* (**B22**)
Impact on parenting techniques	*“To the extent, I have my son, he had an exam* […] *he told me I want to study, and I have a football game. I told him go and play with them; he said what about the exam? I told him go play football* […] *I swear, my wife looked at me and said really? I told her yes! Let him go play football, he will have time to come back and study* […] *he left for 1 h, played a game and came back, took a shower, and studied, the important thing is that he played football”* (**B10**)
Feelings of guilt	*“I get… a breakdown… I lose my nerves… especially that my nephew martyred* […] *I always remember this, when it happened… and I feel that* (he died) *because of me…”* (**A3**)
	*“*(people) *dying in front of* […] (me), *not able to do anything for them… yes war is a tragedy, a tragedy”* (**A5**)
Bereavement	*“I went through a lot of tough circumstances* […]*, honestly my mental state was damaged, and what happened to me is that my mother and both my sisters died, my father is at work and my brother left for college in Beirut and I need to eat and drink and someone to take care of me and help me shower”* (**B8**)
2.3. Financial burden	
	*“And all my teeth fell, now I implanted them. They all fell from here to here, all of them… fell* […] *and it took me 10 years to fix them. I cannot* (afford a one-time payment). […] *3,000 or $4,000”* (**A7**)
	*“The medications no, the ministry of health did not cover them, I buy the medication and then submit it to the NSSF. Some medications are covered by NSSF, and other medications not covered* […] *and even NSSF medications, I would pay for them and throw* […] (the package) *away… without submitting it… you go and submit them and have to wait 6–7 months to get subsidized”* (**A1**)
	*“I even sold my wedding ring to cover* (the cost)*”* (**A17**)
	*“the cost of the medications was what affected me most”* (**A3**)

## 4 Discussion

This study is among the first to briefly shed light on Lebanon’s emergency preparedness, civilian access to care, and the enduring aftermath of injuries from the July 2006 war. By merging our findings with prior research on the political and economic situation, the support provided by the government and humanitarian actors, and the psychological trauma caused by the July 2006 war in Lebanon, improvements could be planned and carried out in preparation for potential forthcoming incidents or conflicts. These improvements could ease the burden on both civilians and the system.

Participants recounted witnessing horrifying and traumatic violence at homes, streets, and hospitals. They detailed their journey to hospitalization and the care received at tertiary centers. While praising Lebanese Red Cross, hospitals, and humanitarian aid, participants noted potential pitfalls that could strain civilians and healthcare. Urgent attention is needed to rectify subpar healthcare, which escalates illness burden and costs (38).

### 4.1 National and health centres’ emergency preparedness

Our study showed that national authorities and the healthcare system in Lebanon are ill-prepared to manage the multidimensional impact of armed conflicts. Hospitals often lacked essential resources, echoing a study published in December 2020 on emergency preparedness that identified similar gaps (39). These deficiencies encompassed surge capacity, surgical care, patient tracking, data storage, recovery protocols, and mental health services. Most of these shortages were prominent in hospitals located in South Lebanon and the Beqaa area, the governorates most affected by the July 2006 war. However, it is worth noting that most hospitals included in that study are located in Beirut and Mount Lebanon, rather than in the areas in question (39). In a highly privatized health care system, despite developing emergency preparedness plans, a study published in July 2022 showed that even private hospitals suffered major challenges during the Beirut Blast in August 2020 (40). This underscores notable shortcomings in the effectiveness of the preparedness plans. However, it is worth nothing that hospitals in Lebanon were struggling financially due to the economic crisis of 2019, which led to shortage of medication, medical equipment, and staff (41). Amidst the financial crisis, the social unrest, the devastating Beirut blast, the ongoing coronavirus pandemic, and outbreaks of diseases such as cholera and measles, the United Nations (UN) declared in May 2023 that the Lebanese health care system is progressively struggling to withstand new shocks (42).

The MoPH in Lebanon claimed to have mobilized donations and provided health facilities in South Lebanon and the Beqaa area with emergency kits and drugs. Unfortunately, road damage and Israeli restrictions hindered aid access, amplifying existing healthcare system inadequacies (18). In times of conflict, healthcare institutions and personnel suffer due to targeting and migration, engendering a detrimental cycle. Concurrently, national authorities lacked robust war response plans, especially in peripheral regions (43). The MoPH’s explanation for medical supply scarcity during the war contradicts effective emergency planning, mirroring a pattern evident after the Beirut blast. Despite the evident major flaws in the government’s response to the 2006 war, a Disaster Risk Management Unit was only established in 2010 – four years post war (44). Unfortunately, this Unit was ill-prepared for potential disasters, as demonstrated by the events surrounding the Beirut blast in 2020. The Beirut explosion disaster emphasized the contributions of citizens, residents, and NGOs, thereby underscoring governmental inadequacies.

These issues emphasize the necessity for comprehensive preparedness strategies and highlight the recurring failure of authorities in crisis situations (45).

### 4.2 Community support

Our findings highlighted that without effective government response, communities turned to each other for assistance. Shared experiences fostered social cohesion and solidarity, prompting community members to aid injured civilians, transcending religious and political divides. Internally displaced persons received support from diverse communities, showcasing a collective identity that helped withstand the atrocities of the war (46). Such neighborly collective coping was observed in other LMIC armed conflicts with limited resources (47).

### 4.3 Provision of medical care during and post-war

Our qualitative study reveals potential shortcomings in initial hospitalization. Patients reported insufficient debridement and ensuing complications, such as infections and chronic pain, aligning with research linking inadequate debridement to infection and delayed amputation (48).

Likewise, our study highlights patients’ restricted access to physical and psychological care, leading to job loss, social isolation, and mental strain. Comparable experiences emerge in other contexts; a study on Kosovar Albanians 6 years after conflict found limited medical access linked to persistent and new PTSD cases (49).

### 4.4 Long-term impact on civilians

Apart from immediate wartime consequences, our findings depict enduring suffering among civilians including physical, psychological, and financial realms. Long-lasting effects include pain, restricted mobility, PTSD symptoms, depression, anxiety, and compromised quality of life. Our results align with Lebanese data displaying elevated PTSD, anxiety, and mood disorders post-July 2006 war (28, 31). Similar patterns manifest in other conflict-affected zones. Gaza’s exposed children exhibited aggression and stress symptoms (12), while adults endured reduced health-related quality of life due to distress and insecurity (50). Comparable literature denotes functional limitations, poor quality of life, and high prevalence of PTSD, depression, and anxiety (51–54).

Beyond direct war injuries, our study emphasises the psychological implications of parental loss for children, linked to symptoms like PTSD, depression, and suicidal thoughts, especially following inadequate care by the surviving parent and their social network (e.g., schools, friends, and neighbors). These findings are consistent with previous studies that linked the death of a parent to increased somatic symptoms, stress, long-term suicide risk, and mortality (55, 56). Unaddressed maternal loss was also associated with adult depression, which emphasises the importance of mothering in a child’s development, often attributed to a more affective and emotional closeness between mothers and children (57–59).

Our research reveals substantial financial burdens on civilians, who must personally fund short- and long-term treatment. Absence of insurance, work compensation, and social support escalates the financial strain on war-injured individuals. Notably, war cost analysis often targets military personnel. A study examining the long-term healthcare requirements of American military service members reported significantly increased healthcare requirements for individuals with combat-related injuries compared to peers with non-combat-related reasons; and therefore, higher healthcare costs per year (60).

### 4.5 Long-term impact on people with disabilities

The narrow confines of masculinity in Lebanese society intensified psychological stress for men grappling with disabilities. Our findings indicate that social and cultural expectations compounded men’s challenges by conflicting with their gender roles. This gender role struggle is not recent and has been documented elsewhere. A study of World War I veterans in Italy explored the impact of normative masculinity on those with physical disabilities, revealing their fears of becoming marginalized and burdensome (61).

Our findings showed that victims with amputations exhibit an additional burden. Their concerns included phantom pain, decreased mobility, unemployment, and the inability to provide for their families. Amputees also suffer from social awkwardness and isolation that stem from amputation taboos, aligning with two studies conducted in the Gaza Strip on conflict-related amputation victims (6, 62).

Though some July 2006 war-disabled civilians received aid, discomfort led to underutilisation of medical devices like prosthetics. This aligns with device dissatisfaction found in studies citing inefficiency, weight, and fit discomfort as reasons for abandonment (63, 64). Improper prosthetic fitting or lack of training by a physical therapist can heighten dissatisfaction (65, 66). Prosthetics, often donated post-conflict in LMICs, may not suit patients, lacking proper training.

### 4.6 Study strengths and limitations

This study represents a pioneering effort to delve into Lebanon’s emergency preparedness, civilian healthcare access, and the lasting physical, psychological, and financial toll inflicted by war injuries during the July 2006 conflict. Our research encompassed victims from various Lebanese regions, primarily those closer to the battlefront. A multi-dimensional perspective was attained through data comparison from medical records and participant interviews, revealing a fragmented healthcare system.

Our study’s contextual specificity is a key limitation. However, our findings echo results from diverse settings, broadening the scope of our recommendations. Another limitation is the selection bias originating from inclusion of only two hospitals. This stemmed from data destruction regulations and limited electronic systems in Lebanon. Consequently, the sample size was small, not capturing all injury types and severities, necessitating further validation. Refusals were more frequent in hospital A, possibly skewing severity representation.

Interpretive bias arose due to absent diagnostic coding and inability to link subsequent treatment records, impacting readmissions and original injuries. Despite efforts, excluding politically affiliated combatants wasn’t entirely definitive. Recall bias emerged as the July war’s time frame surpassed a decade, although forgotten details were supplemented by medical records. Nonetheless, we acknowledge the potential temporal evolution of participants’ perspectives which could have been influenced by factors such as evolving life circumstances, coping mechanisms, and ongoing societal changes. Qualitatively, we focused on participants’ most impactful memories to highlight prominent burdens. However, participants with repressed memories might have opted out, introducing selection bias.

A comprehensive analysis of study challenges and barriers was detailed separately (67).

## 5 Implications and potential recommendations

### 5.1 Emergency preparedness plan

While the MoPH acknowledged delivery hurdles during the 1996 Israeli occupation assault on Lebanon due to shelling (18), it failed to implement crucial measures for prevention, mitigation, and readiness for future crises. The lack of an agile national preparedness plan led to the unavailability or inaccessibility of adequate medical services in certain areas during the July 2006 War. The World Health Organization (WHO) and UN support allowed the MoPH to distribute emergency kits to South of Lebanon and the Beqaa area, one week after the onset of the war (18).

As per the WHO strategic framework, comprehensive preparedness entails actions on community, national/subnational/local, and global/regional tiers (68):

- Community level: pre-emptively equipping outlying areas with first aid training and decentralized medical supplies before the onset of conflict. This ensures the availability of medication and resources even when transportation networks become compromised.
- National/Subnational/Local level: strategies encompass creating public health emergency operations centres, addressing healthcare workforce shortages, and bolstering skill sets. After the July 2006 War, international NGOs, including WHO, facilitated emergency readiness development in Lebanon, potentially valuable post-2020 Beirut Port Explosion (69).
- Global/Regional level: plans include 1- coordinating with multi-sectoral regional and global coordination mechanisms; 2- providing technical assistance and guidance for preparedness, response, and recovery planning; 3- country risk and capacity assessments; and 4- pre-deployment training.

Developing a holistic plan becomes imperative, especially in Lebanon’s dire state—embroiled in a catastrophic economic crisis, a crumbling healthcare system, healthcare professional exodus, and critical resource scarcity.

### 5.2 Support for people with disabilities and amputations

Our study findings highlighted a gap in social and financial support for civilians with disabilities and amputations. Lebanon faces a systematic lack of provisions for rights, resources, and services for persons with disabilities in all areas of their lives. Work and basic services are scarce, inaccessible and of poor quality, especially for employment, education, access to information, participation in public and political life, and law enforcement (70). In 2016, 80% of persons with disability in Lebanon “are not or have never been employed while the legislation establishing an employment quota remained unenforced (71). In a study examining the long-term PTSD prevalence and burden among adult cluster munition victims from the July 2006 war, job instability emerged as the most prevalent socio-economic consequence (88%) (28). In line with the Committee on Economic, Social and Cultural Rights (CESCR), Lebanon must implement the employment quota, fostering decent living standards and career opportunities for individuals with disabilities.

Furthermore, although Lebanon possesses legislation mandating public and private entities to facilitate accessibility for individuals with disabilities using essential engineering and equipment, the government’s inertia, seen in its failure to issue application decrees and carry out requisite procedures, has hindered execution and enforcement. Moreover, the State and involved entities missed an opportunity to integrate inclusivity during the post-July 2006 war reconstruction, failing to render rebuilt infrastructure and buildings accessible and inclusive from inception (70). Hence, it is imperative for the Lebanese government to actively enforce the mentioned legislation.

As documented by past research, people with disabilities, including those who became disabled during wars, face negative and discriminatory attitudes. Good fit prosthetics and other assistive devices as well as individualized rehabilitation are vital for people with disability to increase their mobility and give them a sense of dignity (72). Raising awareness about disability is essential to foster respect for the rights and dignity of people with disability and to promote awareness of their capabilities and contributions (73).

Furthermore, disability threatens the societal concept of “normative” masculinity as it is often represented as emasculation due to its association with dependence, weakness, and vulnerability (74, 75). The demands of a competitive labor force, cultural cults of hyper-masculine ableism, and social expectations of men as independent and strong breadwinners further increase the burden on people with disabilities as they no longer fit the “norm” (74). It is therefore also important to challenge and confront the gender role conflict to reduce its harmful influence on people, including the war injured.

### 5.3 Improving the research culture

The limitations of this study should be considered to improve the research culture in LMICs and in conflict settings. Only seven institutions in Lebanon conduct health policy and systems research (HPSR): six in Beirut and one in Mount Lebanon (76). It would be beneficial for more hospitals to incorporate research centres in their departments and to emphasize the importance of developing research skills and of having an electronic health system. Improving the research culture and decentralizing it is a critical step prior to conducting similar research. More detailed recommendations were previously published (67).

## 6 Conclusion

Civilians in LMICs are vulnerable to many of the atrocities of wars. We identified several gaps on the macro- and micro-levels of policy, healthcare, and society in the context of the war in Lebanon, all of which need to be addressed for better treatment and recovery of injured civilians. Due to the lack of an agile national emergency preparedness plan, injured civilians, especially those outside the capital and major cities, were faced with inadequate access to high-quality healthcare. Consequently, affected civilians suffer from a long-term physical, psychological, and financial burden. This study highlights the need for the improvement, training on, and implementation of emergency preparedness plans at the national, local, and tertiary care centre levels. It also emphasises the need for a targeted approach in peripheral hospitals by training healthcare personnel on conflict trauma. Finally, there is a need for the initiation and implementation of occupational therapy, affordable psychosocial counseling, and national campaigns to abolish the stigma on gender roles, amputation, and disabilities.

## Data availability statement

The original contributions presented in the study are included in the article/Supplementary material, further inquiries can be directed to the corresponding author/s.

## Ethics statement

The studies involving humans were approved by the Institutional Review Board at the American University of Beirut (SBS-2019-0226). The studies were conducted in accordance with the local legislation and institutional requirements. The ethics committee/institutional review board waived the requirement of written informed consent for participation from the participants or the participants’ legal guardians/next of kin because the research posed minimal risk of harm to subjects since interviewers were adequately trained in the appropriate approach to patients and inquiring about the necessary questions during interviews without inducing emotional distress. Additionally, having their signatures on paper could have caused distress to subjects, especially in light of the study’s political nature. Written informed consent was not obtained from the individual(s) for the publication of any potentially identifiable images or data included in this article because obtaining subjects’ signatures on paper could have caused distress due to the political nature of the study. Therefore, oral consent was sought instead, and only a minimal data set has been published.

## Author contributions

EK: Conceptualization, Data curation, Formal analysis, Investigation, Methodology, Visualization, Writing – original draft, Writing – review & editing. MM: Conceptualization, Funding acquisition, Methodology, Project administration, Writing – review & editing. GH-A: Formal analysis, Methodology, Writing – review & editing. NE: Formal analysis, Writing – review & editing. ZA-S: Project administration, Writing – review & editing. TF: Investigation, Visualization, Writing – review & editing. DM: Investigation, Writing – review & editing. MH: Investigation, Writing – review & editing. RA-K: Conceptualization, Writing – review & editing. BC: Investigation, Writing – review & editing. AE: Investigation, Writing – review & editing. SH: Conceptualization, Writing – review & editing. GA-S: Conceptualization, Funding acquisition, Methodology, Writing – review & editing.

## Glossary

AMT: Abbreviated Mental Test
CESCR: Committee on Economic, Social, and Cultural Rights
GPCOG: General Practitioner Assessment of Cognition
GRAMMS: Good Reporting of A Mixed Methods Study
HPSR: Health Policy and Systems Research
IRB: Institutional Review Board
LMIC: Low- and Middle-Income Country
LRC: Lebanese Red Cross
MENA: Middle East and North Africa
MoPH: Ministry of Public Health
NGO: Non-Governmental Organization
NSSF: National Social Security Fund
PLO: Palestine Liberation Organization
PTSD: Post-Traumatic Stress Disorder
UN: United Nations
WHO: World Health Organization.
