# Supplementary material for: Long-term burden of war injuries among civilians in LMICs: case of the July 2006 war in Lebanon
Source: Front Public Health. 2023 Dec 8;11:1305021. doi: 10.3389/fpubh.2023.1305021 (PMC10748398; doi:10.3389/fpubh.2023.1305021)
Supplement: Supplementary file 1 [file Table_1.DOCX]

Supplementary Material

# Supplementary Data 1: Topic Guide

# Qualitative Interview Semi-structured Questionnaire

[Principal Investigator Dr. Ghassan Abu-Sitta]

Hello, My name is X. Thank you for consenting to participate in the study. I am conducting a research study on the burden of war injuries that the July 2006 war has caused. I would like to reaffirm that all of your answers will be treated with confidentiality and anonymity and will not be shared beyond the research team.

**Introduction:**

This is an IRB approved study (as mentioned in the informed consent form). We reviewed all of the charts of the patients injured during the July War 2006 in Lebanon who presented to hospital X and we found that you were among the injured civilians affected by this war.

**Patient characteristics:**

1. Age
2. Marital Status
3. Offspring
4. Dependents
5. Education
6. Occupation

**Interview guide:**

Description of the situation

1. Can you please tell us more about what happened the day of the injury?
   1. What was the mechanism of injury (missile, bullet…)?
   2. What parts of your body were affected?
   3. Where were you at the time? Were other members of the family injured? If yes whom and what kind of injury…
   4. Was the building you were in completely destroyed?
2. Do you remember what hospital you were transferred to first?
3. How did you get to the hospital?
4. How long did you stay in the first hospital?
5. What surgical procedures were operated on you?
6. Were you transferred directly to hospital X or first to another hospital?
7. How long did you stay in the hospital before being transferred to hospital X?
8. When were you transferred to hospital X?
9. What was the first surgery you got?
   1. Which hospital?
   2. How about further surgeries? How many did you get in total? Which hospitals?
   3. Did you have to return to the hospital for further surgeries? When? How many times? What was done?
   4. After hospital X, did you have other surgeries related to your injury? When and where?
10. Did you have to go through amputations? Do you have prosthesis?
    1. How many times have you had surgery for your prosthesis?
    2. How many times have you had your prosthesis changed?

Burden

Financial cost

1. Let us talk about the cost(s) of the surgery(ies) you had?
2. What was the financial cost for each surgery?
3. The cost of your treatment with medications etc..?
4. Who paid?
5. What about other costs? For example, rehabilitation? Prosthesis?
6. Did you get any other support from an NGO or the government?
7. If yes, what kind of help?
8. Was this covered by the insurance/ministry, government, out-of-pocket etc...?

Non-financial cost

1. What happened to your work/education situation after injury?
   1. Were you able to work/study after you recovered? How long was your work/study disrupted for? (after each surgery)
   2. If yes, how much time after the injury were you able to return to work/study?
   3. How did this impact the quality of your life? (pain, general health, mobility, mental health, permanent disability…)
   4. How did this impact the quality of life of your dependents?
   5. Were you dependent on anyone throughout the treatment process and for how long?
   6. Who took care of you during the treatment process and after?
   7. Did the caregiver continue his/her work or had to stop in order to help you?
   8. If yes, how did the life of the individuals that were dependent on you get affected by the injury?

Long-term impact of injury

1. Do you still have any remaining disabilities from the injury?
   1. If yes, what aspect of your life did it mostly affect (social, financial, functionality…), can you elaborate more?

Conclusion

Is there anything you would like to add?
